# COVID-19 Inflammatory Markers and Vitamin D Relationship in Pediatric Patients

**DOI:** 10.3390/life13010091

**Published:** 2022-12-28

**Authors:** Iulia Cristina Bagiu, Ileana Luminita Scurtu, Delia Ioana Horhat, Ion Cristian Mot, Razvan Mihai Horhat, Radu Vasile Bagiu, Ionut Dragos Capraru, Mircea Mihai Diaconu, Ovidiu Adam, Bogdan Ciornei, Dan Dumitru Vulcanescu, Iulius Juganaru, Andrei-Cristian Bondar, Florin George Horhat

**Affiliations:** 1 Emergency Hospital for Children “Louis Turcanu”, 300011 Timisoara, Romania; 2 Department of Microbiology, Multidisciplinary Research Center on Antimicrobial Resistance, “Victor Babes” University of Medicine and Pharmacy, Eftimie Murgu Sq. No. 2, 300041 Timisoara, Romania; 3 Faculty of Medicine, Vasile Goldis Western University of Arad, Rebreanu 86, 310414 Arad, Romania; 4 ENT Department, “Victor Babes” University of Medicine and Pharmacy, Eftimie Murgu Sq. No. 2, 300041 Timisoara, Romania; 5 3rd Department, Discipline of Odontotherapy and Endodontics, Faculty of Dental Medicine, TADERP Research Center, “Victor Babes” University of Medicine and Pharmacy, Eftimie Murgu Sq. No. 2, 300041 Timisoara, Romania; 6 Department of Hygiene, Preventive Medicine Study Center, “Victor Babes” University of Medicine and Pharmacy, Eftimie Murgu Sq. No. 2, 300041 Timisoara, Romania; 7 Department of Parasitology, “Victor Babes” University of Medicine and Pharmacy, Eftimie Murgu Sq. No. 2, 300041 Timisoara, Romania; 8 Department of Obstetrics and Gynecology, “Victor Babes” University of Medicine and Pharmacy, Eftimie Murgu Sq. No. 2, 300041 Timisoara, Romania; 9 Department of Pediatric Surgery and Orthopedics, “Victor Babes” University of Medicine and Pharmacy, Eftimie Murgu Sq. No. 2, 300041 Timisoara, Romania; 10 Department XI, First Discipline of Pediatrics, “Victor Babes” University of Medicine and Pharmacy, Eftimie Murgu Sq. No. 2, 300041 Timisoara, Romania; 11 Psychiatry Hospital “Prof. Dr. Alexandru Obregia”, Soseaua Berceni 10, 041914 Bucuresti, Romania

**Keywords:** COVID-19, SARS-CoV-2, creatine kinase, lactate dehydrogenase, C reactive protein, vitamin D, children

## Abstract

Background: Biomarkers play an important role in COVID-19, and more research in this regard is needed, especially in the case of children. This study aimed to look for a link between the C reactive protein (CRP), lactate dehydrogenase (LDH), creatine kinase (CK), vitamin D and COVID-19 in pediatric patients. Methods: This is a retrospective cohort study, performed on children diagnosed positively with COVID-19 at a children’s hospital in western Romania. Available CRP, LDH, CK vitamin D and clinical severity were recorded. For each biomarker, groups were formed by patients’ age. Mean/median group differences were assessed using Student’s *t* test or Mann–Whitney and Kruskal–Wallis with Dunn’s post hoc tests. Association was assessed using the chi^2^ test, while correlation was assessed using Spearman’s rank correlation. Results: 181 positive children were studied between 1 August 2021 and 1 February 2022. Average age was 8.76 years (SD = 3.93). There were 94 (51.93%) males and 87 (48.07%) females. The cases were: 62 asymptomatic (34.25%), 107 mild (59.12%), 9 moderate (4.97%), 3 severe (1.66%). Regarding CRP, a significant difference between older and younger patients was observed (*p* = 0.0034). Clinical severity was associated with CRP (*p* = 0.0281), LDH (*p* = 0.0410) and vitamin D (*p* = 0.0444). Regarding CK, no differences or associations proved significant. Correlation testing was conducted for CRP, LDH, vitamin D and clinical signs. With the exception of LDH-CRP and LDH-vitamin D, all relationships proved statistically significant. Conclusions: CRP, LDH and vitamin D levels are important biomarkers for COVID-19-positive pediatric patients, while CK was mostly within normal ranges.

## 1. Introduction

The first coronavirus to impact humankind on a large scale was SARS-CoV (severe acute respiratory syndrome coronavirus), which caused a pandemic in 2003. Ten years later, MERS-CoV (Middle East respiratory syndrome coronavirus) caused a major epidemic in the Middle East and was declared an international medical emergency leading to the deaths of hundreds of people [1]. From the literature, it can be seen that from 2003 until now different types of coronaviruses have been identified, both in animals and in humans. This type of virus can be easily transmitted from one person to another, causing infections of varying severity, mostly common colds, but also more serious infectious diseases [1,2].

At the end of 2019, a new coronavirus (2019-nCoV) was identified in China, causing the infectious disease Coronavirus 2019, later called COVID-19 and declared a global medical emergency by the WHO (World Health Organization) [2,3]. Later, the name of the virus was changed to SARS-CoV-2 (severe acute respiratory syndrome coronavirus 2). As of March 2020, due to the global spread and severity of the COVID-19 disease, the WHO has defined the outbreak of COVID-19 as a pandemic. COVID-19 infection is manifested by severe acute respiratory syndrome, which results in the admission of many patients, especially elders and people with comorbidities such as diabetes, heart or lung conditions, into intensive care units (ICU) [4,5].

The LDH marker characterizes the following inflammatory diseases: myocardial infarction, cancer and cardiopulmonary diseases, and it is a potential marker of vascular permeability in immune-mediated lung lesions. Shanghai health authorities said in March 2020 that LDH could also be considered as a relevant predictive marker for the early identification of lung damage, and therefore severe cases of COVID-19 [6].

The CRP is also a biomarker that defines inflammatory diseases and traumas [6,7]. In a Turkish study on adult patients, it was shown that both LDH and CRP were associated with lung lesions in the early stages of COVID-19 disease, reflecting the severity of the disease and also possibly reflecting the need for chest CT (Computed Tomography) [5]. As for CK, Song noted in his study that an increase in the value of this biomarker can reduce the survival rate of patients; in other words, they can predict mortality [8].

Although the COVID-19 infection appeared in 2019, Laila Bourkhissi stated in September 2020 that little was known about this infection in children [9]. In the pediatric population, statistics showed that the COVID-19 disease is less fatal, however these statistics could be influenced by various other factors. In the pediatric population, there are situations when both biomarkers and the results of radiological investigations overlap with other viral infections; therefore, an algorithmic diagnosis should replace the dependence on a single diagnosis and screening test [10].

For example, in the case of the CK marker, a retrospective study conducted in early 2021 over a two-month period at the Wuhan Children’s Hospital concluded that elevated serum CK values could indicate lesions in several organs and an increased immune response in children with COVID-19. Thus, additional monitoring of this marker would help clinicians in choosing a more suitable medication for patients with COVID-19 [11,12].

Vitamin D (25-hydroxycholecalciferol–25-OHD) deficiency is a major health problem. It is a pluripotent hormone with important connections to the immune response. Yet, it is estimated that more than one billion people worldwide have vitamin D deficiency [13]. Its role in viral infections is important, as it induces cathelicidins and defensins, with the ability to reduce viral replication rate. Moreover, it promotes anti-inflammatory cytokine production [14]. A few studies have suggested a link between symptomatology, severity and outcomes in regard to concentrations of vitamin D [15,16,17].

Moreover, alongside inhibiting hyperinflammatory reactions, vitamin D accelerates the healing process in the affected areas, especially in lung tissue. Moreover, vitamin D deficiency has been associated with the severity and mortality of COVID-19 cases, with a high prevalence of hypovitaminosis D found in patients with COVID-19 and acute respiratory failure [18].

## 2. Materials and Methods

### 2.1. Study Design and Participants

This study was designed as a retrospective cohort study, performed on children diagnosed positively with COVID-19 using a RT-PCR molecular test (reverse transcription polymerase chain reaction), upon admission to the emergency reception unit (ER) at the Pediatric Hospital “Louis Turcanu” in Timisoara between 1 August 2021 and 1 February 2022 (6 months) in order to assess proinflammatory biomarkers in the prognosis of COVID-19 disease. The criteria for admission of patients to the study were: positive COVID-19 RT-PCR test, existing test results for CRP, LDH, CK and/or vitamin D.

Patients excluded from the study were COVID-19-positive patients without CRP, LDH and CK data. During the studied period, 849 children were tested in the ER. A total of 181 patients were enrolled in the study, as the results from the COVID-19 testing came back positive. One hundred and fifty-two patients presented data for CRP analysis, while only 44 patients presented data for LDH analysis and 36 patients for CK analysis. For each analysis, the patients were split into age groups. For CRP and LDH there were two groups, one containing patients under 1 year old and one containing patients over 1 year old (maximum was 17).

For the analysis of CK, the patients were divided into four groups, depending on age: group 0–4 years, group 4–7 years, group 7–13 years and group 13–17. The older children’s groups were then divided depending on sex into male and female. This division was performed in regard to the normal values according to age for children between 0 and 7 years and according to age and sex for those aged between 7 and 17 years.

The severity of the disease was classified as asymptomatic, mild, moderate, severe and critical according to the clinical characteristic and laboratory results based on the existing data [19,20]: (1) asymptomatic cases—with a positive RT-PCR test and without any clinical findings; (2) mild—with upper respiratory tract infection symptoms including low-grade fever, fatigue, myalgia, cough and sore throat, without pneumonia; (3) moderate cases—defined by pneumonia with complaints of fever and cough but without the symptoms dyspnea and hypoxemia and without the need for oxygen; (4) severe cases—with fever and cough in the early period which develop dyspnea and central cyanosis within a week (arterial oxygen saturation of <92%); and (5) critical cases—which develop acute respiratory distress or respiratory failure rapidly, and who tend to develop shock, encephalopathy, myocardial affection, coagulation dysfunction and acute kidney injury.

### 2.2. Data Collection

Since this study has a retrospective cohort character, the patients’ written consent was not required, as the data were obtained from the computer register of the laboratory. Epidemiological information such as age, sex and diagnosis were also assessed. Identification data of the patients such as names, id numbers or contact information was not collected for the present study.

The determination of the biomarkers was performed upon admission to the ER for CRP, LDH, CK and vitamin D, according to existing guidelines [21,22,23,24,25], and compared with the corresponding normal values for each biomarker as follows (Table 1): CRP (<5 mg/L); LDH (0–1 years 225 U/L–600 U/L, 1–17 years 120 U/L–300 U/L); CK (0–4 years: 24 U/L–228 U/L, 4–7 years: 24 U/L–149 U/L, F: 7–13 years: 24 U/L–154 U/L, 13–17 years: 24 U/L–123 U/L, Male 7–13 years: 24 U/L–247 U/L, 13–1724 U/L–270 U/L), 25-OHD (>30 ng/mL), as described in Table 1. All cut-off values followed the normal range of the laboratory where the study took place, in accordance with the existing guidelines. Data on the COVID-19 results obtained from laboratory tests were also reviewed.

### 2.3. Laboratory Testing

A fully automated clinical chemistry instrument, Cobas Integra 400 plus (Roche Diagnostics), was used for analyzing the serum sample. Nasopharyngeal swabs of the patients were examined using the BIO-RAD CFX96 Real-Time System C1000 Touch Thermal Cycler Device.

### 2.4. Statistical Analysis

Statistical analysis was performed using the statistical program GraphPad Prism 5 (GraphPad Software, San Diego, CA, USA). All continuous variables were assessed with the Kolmogorov–Smirnov test to check for normal distribution. Continuous variables with a normal distribution were expressed as mean and standard deviation (SD) and were compared using a two-sample *t*-test. Non-parametric variables were expressed as median and interquartile range (IQR) and were compared using the Mann–Whitney and Kruskal–Wallis with Dunn’s post hoc tests. In order to find associations between the existence of clinical respiratory signs and the elevated status of the studied biomarkers, contingency tables were created and analyzed using the chi^2^ test. Afterwards, links between biomarkers levels were assessed using Spearman’s rank correlation. For all tests, *p*-Value (*p*) <0.05 was considered statistically significant.

## 3. Results

A total of 849 children were tested during the study period. Of these, 181 children were found to be positive for COVID-19 upon admission to the ER and were enrolled in the study. Of the total number of admitted patients, 25.4% were aged between 0 and 1 year, and 74.6% were older than one year, with an average age of 8.76 (SD = 3.93) years. There were 94 (51.93%) males and 87 (48.07%) females.

Regarding clinical presentation, most patients were asymptomatic (*n* = 62, 34.25%) or had mild symptoms at the time of admission to the ER (*n* = 107, 59.12%). There were 12 more serious cases, of which 9 (4.97%) were moderate and 3 (1.66%) were severe and were further hospitalized in the ICU. Most moderate cases were hospitalized for less than two weeks (median: 12.25 days, IQR = 15.5), while the severe cases were admitted to the ICU for more than two weeks (median: 24, IQR = 5). Only one case (0.55%) was critical, with a period of about 30 days of hospitalization to the ICU, but with a favorable evolution. No patient had succumbed during our study.

The most common symptoms reported included fever (*n* = 61, 51.38%), cough (*n* = 31, 25.97%), sore throat (*n* = 22, 18.23%), fatigue (*n* = 21, 17.68%) and muscle and headaches (*n* = 19, 16.02%). Of the 12 more serious patients, observed clinical signs at presentation at the ER, as follows: six presented with nonspecific pneumonia, two with hypertension, two with acute laryngitis, one with acute rhinopharyngitis, one with Crohn’s disease in treatment, one with Type 1 Diabetes, one with neonatal jaundice. Of those with pneumonia, three were hospitalized in the ICU.

### 3.1. C Reactive Protein Analysis

**Figure 1:**
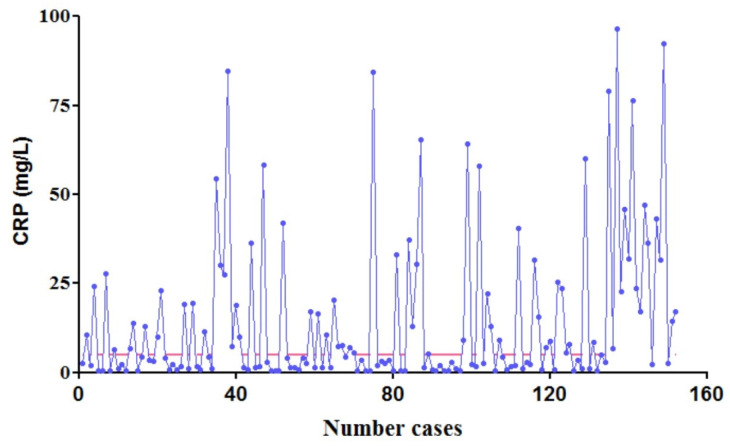
Patient CRP values (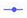 CRP values determined, 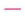 maximum normal CRP, 5 mg/L).

The total number of patients with available data regarding CRP was 152. Their results can be seen in Figure 1. Although normal values (5 mg/L) of this biomarker do not depend on age and sex, patients were divided into two groups, one including patients under 1 year and one including children over 1 year, in order to assess a more accurate view of the role of this protein in the COVID-19 disease in regard to the pediatric population.

**Figure 2:**
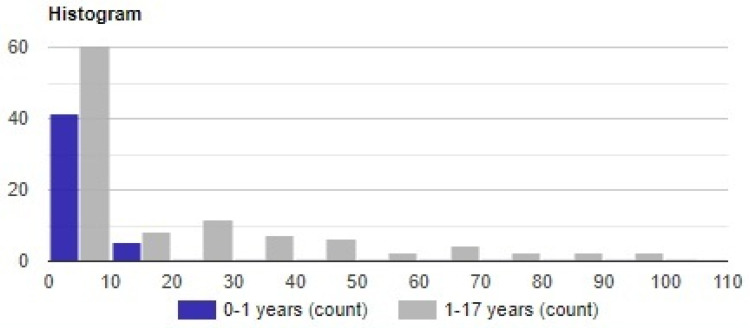
Histogram with the frequency of case distribution according to CRP value.

The group consisting of children under 1 year showed a median CRP value of 3.26 mg/L (IQR: 6.27), while the older group’s median was 4.83 mg/L (IQR: 25.54). This difference proved statistically significant as the *p*-Value was 0.0034. This difference can be graphically observed in the plotted histogram present in Figure 2.

There were 81 (53.29%) male and 71 (46.71%) female patients. Median value for males was 4.32 (IQR = 16.52), while for females was 4.26 (IQR = 16.32). The result of the Mann–Whitney test returned a *p*-Value of 0.4471, resulting in no significant differences when it comes to sex.

Regarding clinical status of patients with available CRP values, there were 55 (36.18%) asymptomatic, 86 (56.58%) with mild symptoms, 8 (5.26%) with moderate symptoms and the 3 (1.97%) more severe cases. A chi^2^ test revealed a *p*-Value of 0.0281 when it came to the association between clinical severity and CRP levels.

For the three patients with pneumonia who were admitted to the ICU, the mean CRP value on the first day was 12.23 mg/L. Afterwards, on day 5 of hospitalization it reached a maximum of 104.5 mg/L, which after treatment decreased to 43.10 mg/L.

### 3.2. Lactate Dehydrogenase Analysis

Of the total number of patients who presented to the ER and were positively diagnosed with COVID-19, LDH levels were determined in only 44 patients.

**Figure 3:**
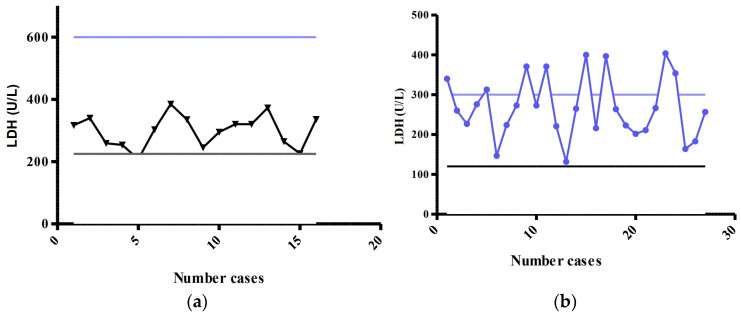
LDH values for: (**a**) group 0–1 year: 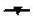 determined LDH, 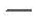 minimum normal value, 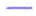 maximum normal value; (**b**) Group 1–17 years: 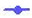 determined LDH, 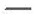 minimum normal value, 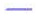 maximum normal value.

The patients enrolled in the study were divided into two groups according to age, group 0–1 year (16 patients) and group 1–17 years (28 patients). From the graphs in Figure 3 we can see that the serum LDH values measured in the age group 0–1 year are much more compact, although the size of the interval between the minimum and maximum normal value is about 400 U/L, compared to serum values measured in the age group 1–17 years.

**Figure 4:**
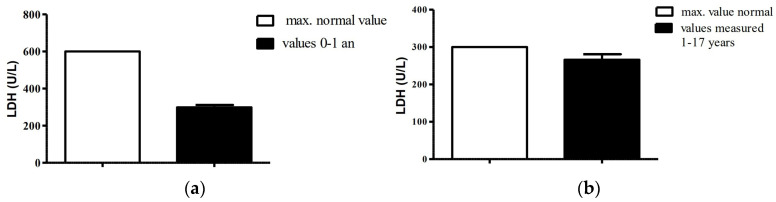
Determined LDH values compared to normal values: (**a**) group 0–1 year; (**b**) group 1–17 years.

LDH values for children aged 0–1 years measured in the laboratory were within normal limits, with an average of 299.13 U/L (SD = 57.78), which is half of the maximum normal value (600 U/L). Although there were LDH values above the maximum normal value for the age group 1–17 years, the average was 270.18 (SD = 81.37) (Figure 4). When testing for differences using a two-sample *t* test, no statistically significant differences were observed as *p* = 0.1777.

Regarding the sex distribution, there were 21 males (47.73%) and 23 females (52.27%). Mean values were 286.10 (SD = 62.55) for boys and 275.78 (SD = 84.73) for girls. Regarding clinical aspects, there were 12 (27.27%) asymptomatic patients, 22 (50%) with mild symptoms, 7 (15.91%) with moderate symptoms and the 3 more severe cases (6.82%). A chi^2^ test revealed a *p*-Value of 0.0410 when it came to the association between clinical severity and LDH levels.

From the 44 children, 6 showed more serious clinical signs at admission to the ER, including the patients who required ICU admission. Five were diagnosed with pneumonia, two with hypertension, one with rhinopharyngitis and one child known to have type I diabetes. Measured serum LDH values were increased in patients with clinical signs of respiratory problems.

### 3.3. Creatine Kinase Analysis

**Figure 5:**
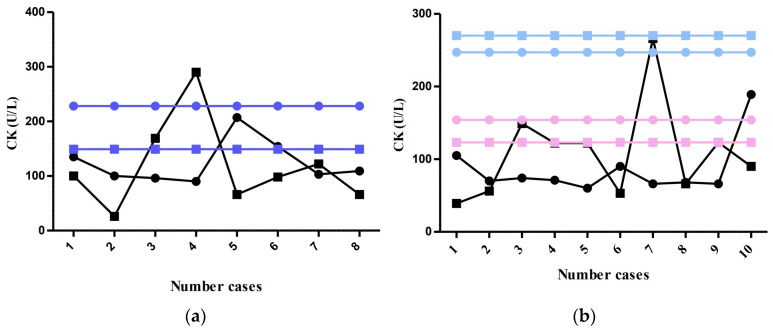
Determined CK values: (**a**) group 0–7 years: 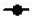 0–4 years, 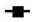 4–7 years, 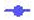 maximum normal value for 0–4 years, 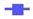 maximum normal value for 4–7 years; (**b**) group 7–17 years: 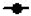 7–13 years, 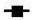 13–17 years, 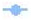 7–13 years maximum normal value for males, 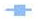 13–17 years maximum normal value for males, 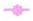 7–13 years maximum normal value for females, 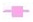 13–17 years maximum normal value for females.

For the analysis of CK, data for 36 patients were available, aged between 0 and 17 years old, that were reviewed in Figure 5. These patients were divided into four groups, depending on age: group 0–4 years, group 4–7 years, group 7–13 years and group 13–17. The older children’s groups were then divided depending on sex into male and female.

This division of patients into four groups was conducted to assess the normal values according to age for children between 0 and 7 years and according to age and sex for those aged between 7 and 17 years.

For the first two groups, 16 patients were enrolled in the study: 8 patients per group, with a median of 99.5 (IQR = 37.5) for group 0–4 years and a median of 108 (IQR = 30) for group 4–7 years. The older children’s groups contained 10 patients per group, with a median value of 82 (IQR = 12.5) for group 7–13 years and 108.5 (IQR = 59.25) for group 13–17. The result of the Kruskal–Wallis test was *p* = 0.1677. Moreover, for the older children’s subgrouping according to sex, there were 50% males and 50% females. The boys’ group median was 81 (IQR = 49), while the girls’ group was 91 (IQR = 28). The Mann–Whitney test returned a *p*-Value of 0.5966 with no significant differences.

Regarding clinical status of patients with available CK values, there were 10 (27.78%) asymptomatic, 17 (47.22%) with mild symptoms, 6 (16.67%) with moderate symptoms and the 3 (8.33%) more severe cases. A chi^2^ test revealed a *p*-Value of 0.4352 when it came to the association between clinical severity and CK levels.

Out of the total number of patients studied, five patients presented at the ER with clinical signs of pneumonia, two with acute laryngitis and one with neonatal jaundice. One patient was in a serious condition and was admitted to the ICU. The level of serum CK was normal in all five patients with pneumonia, while only in one patient with acute laryngitis the value was elevated.

Moreover, in this study we tried to find a link between the increased levels of values of the studied biomarkers in regards to the clinical respiratory signs of patients. This led to the creation of contingency tables for each biomarker, which were then tested using chi^2^. This can be seen in Table 2.

### 3.4. Vitamin D Analysis

Of the total number of patients who presented to the ER and were positively diagnosed with COVID-19, vitamin D levels were determined in only 56 patients. Overall, the median value was 20.21 (IQR = 21.83), which shows that most patients had lower levels of vitamin D. The patients were divided into two groups according to age, group 0–1 year (22 patients) and group 1–17 years (34 patients). Median value for the younger group was 17.81 (IQR = 19.17), while for the older group it was 20.99 (IQR = 19.80). This difference was not statistically significant at *p* = 0.1398. There were 31 (55.36%) males and 25 (44.64%) females. Median value for males was 22.17 (IQR = 23.07) and 19.41 (IQR = 15.59) for females. The *p* value was 0.9409.

Regarding clinical aspects, there were 17 (30.36%) asymptomatic patients, 23 (42.86%) with mild symptoms, 12 (21.43%) with moderate symptoms and the 3 more severe cases (5.36%). A chi^2^ test revealed a *p* value of 0.0444 when it came to the association between clinical severity and vitamin D levels.

### 3.5. Correlation Analysis

Considering that no CK association could be evidenced, a further link between CRP, LDH, vitamin D values and the existence of clinical symptoms was assessed using Spearman’s rank correlation test. A total of 27 out of 181 patients had available data on all three variables (Table 3).

The results of the correlation test are presented in Table 4. Regarding the CRP levels and the existence of clinical signs, a non-significant moderate direct correlation was observed. The link between CRP and LDH was observed as non-significant and direct, yet weak. However, the link between CRP and vitamin D proved to be significant, strong and indirect.

The relationship of LDH levels with clinical signs was significant, strong and direct. On the other hand, the relationship of LDH with vitamin D could be described as significant, moderate and indirect. Lastly, similar values were observed regarding the relationship between vitamin D levels and clinical signs, resulting in a significant moderate indirect relationship.

## 4. Discussion

Early in the pandemic, only a small number of cases of COVID-19 in children was reported, but as time went on, more cases were observed. Rapid identification and provision of appropriate means of treatment are crucial factors in the management of patients with COVID-19 to prevent complications.

In adults, there are many therapeutic approaches, which include agents with direct action such as antivirals (remdesivir, favipiravir, lopinavir/ritonavir, molnupiravir, nirmatrelvir/ritonavir, simeprevir), antimalarials (chloroquine, hydroxychloroquine), antibiotics (azithromycin), or with supportive action, such as interleukin inhibitors (anakinra, tocilizumab), Janus kinase inhibitors (baricitinib), corticotherapy (dexamethasone), anticoagulants (low molecular weight heparin) and non-steroid anti-inflammatory drugs. Information about treatment options for children is somewhat scarce, however it is known that remdesevir and nirmatrelvir/ritonavir were approved for use, while the use of lopinavir/ritonavir was discouraged in pediatric patients [26,27].

Regarding clinical presentation, the number of asymptomatic children was within the known range of around a third of the total patients, as described by previous studies, such as Ylimaz et al. [14], Say et al. [28] or Jat et al. [29].

It was documented that CRP values have a predictive role in regard to severity in adults [30,31]. Usually, this is due to how the immune system responds to the SARS-CoV-2 infection, which can trigger a cytokine storm. This massive release on pro-inflammatory cytokines can cause acute lung injury, resulting in an unfavorable prognosis, especially in elders [32].

The results of our study show a statistical significance of cases with increased CRP value, which can be associated with clinical severity. It is also important to note that this biomarker was higher in older children and adolescents, whose immune systems are more developed. In our study, there were nine cases with moderate and three with severe symptomatology. Most moderate cases were hospitalized for less than two weeks, while the severe cases were admitted to the ICU for more than two weeks. Only one patient was critical and was hospitalized in the ICU for 30 days. Eight of the moderate cases and all three severe patients were older than 1 year and had elevated CRP values with the exception of two moderate cases.

Following a retrospective analysis of a group of 3424 children in Indonesia, it was concluded that CRP levels may be a good indicator for the treatment and early identification of the disease in children [33].

LDH implication requires further studies to indicate how relevant this marker is in assessing the severity of COVID-19 disease, as sometimes data on this biomarker ended up being contradictory [34,35]. In our study, children under 1 year had normal LDH values, while eight of the older children had elevated levels. There was no difference between the two groups’ means, yet the upper normal level differs in regard to age (group 0–1 years: 600 U/L, group 1–17 years: 300 U/L). Moreover, the eight older children with elevated LDH included the one child admitted to the ICU, the other two with severe symptomatology and another five patients with moderate symptomatology.

LDH levels have been associated with the clinical severity since the beginning of the pandemic. Although controversial, many authors agree on and encourage its use as a potential clinical biomarker [36,37]. A study by Du et al. even suggests that serum LDH in pediatric cases were more significantly increased than in adults [38]. As such, more attention should be paid to this biomarker in children older than 1 year.

During our study period, none of the patients died. Although the number of cases with clinical signs is small, it can be seen that elevated CRP and LDH may be associated with the severity of the disease. For initial appropriate treatment in the early stages of the disease, CRP levels can be used to identify patients with early-stage COVID-19 at risk of severe disease development and the risk of hospitalization, especially in older children. Links between CRP and LDH proved weak and non-significant. However, CRP presented a significant moderate link in regard to clinical manifestations.

CK levels are usually associated with COVID-19 in adults [39]. In children, however, this biomarker remains controversial, as several studies have reported elevated values [40,41] while other have reported normal values for this biomarker [42,43]. In our study, no association could be observed between this biomarker and the severity of cases and, as such, it was not researched further.

Vitamin D plays an important role in the modulation of immune response [15]. Other authors have raised the question in regard to its use as a predictive factor [16,44]. Peng et al. have assessed that children with vitamin D insufficiency might have poorer clinical outcome in the Omicron subvariant BA 2 infections [45]. Our study showed that patients with lower vitamin D had an indirect relationship with CRP and LDH values, and with clinical existence of symptoms and severity.

The link between vitamin D and CRP values is still up to debate, as a study by Alpcan et al. found that in their patients the differences were not statistically significant (*p* = 0.074), close to the breakpoint of statistically significance [13]. On the other hand, a study by Daneshkhah Aet et al. suggested otherwise [45]. In our study, a link between vitamin D and CRP proved significant, strong and indirect. Moreover, the relationship between vitamin D and LDH also proved significant and indirect, yet weak.

Moreover, in a study by Michael et al., supplementation of vitamin D in adults and elders proved to decrease the risk of mechanical ventilation and clinical severity in COVID-19 [46]. This is important, as studies by Lee et al. and Beyazgul et al. concluded that during the pandemic, the vitamin D levels of children were lower than before the pandemic, especially in younger children [47,48]. In our study, vitamin D levels were lower in younger children as well. As such, it is important to start vitamin D supplementation in children. This point is supported by a more recent randomized controlled trial done by Zurita-Cruz et al. [49].

### Limitations

As all retrospective studies, this article presents several limitations. First and foremost, the sample size was small and data distribution was somewhat heterogeneous, as not all patients had available data for all studied biomarkers. Therefore, the statistical power is reduced, increasing the risk of statistical error. Moreover, data was missing in regard to pediatric multi system inflammatory syndrome, which is a serious condition sometimes linked to COVID-19, suggesting that future studies should include this disease manifestation.

## 5. Conclusions

In the present study, it was observed that CRP, LDH and vitamin D levels should be taken into account as biomarkers for COVID-19-positive pediatric patients. They were also associated with respiratory signs and symptoms such as pneumonia, rhinopharyngitis and laryngitis. However, CK values were mostly within normal ranges and cannot be considered as biomarker in the assessment of the health of pediatric patients with COVID-19. Moreover, CRP and LDH levels were higher in children older than 1 year, which prompts pediatricians to be on the lookout for these markers when dealing with older children and adolescents. Vitamin D also proved useful, as it showed an indirect moderate link with CRP and LDH levels, as well as clinical presentation and symptomatology, relationships that proved statistically significant.

## Figures and Tables

**Table 1:** Normal range of laboratory values distributed by age and sex.

	CRP (mg/L)	LDH (U/L) 0–1 Years	LDH (U/L) 1–17 Years	CK (U/L) 0–4 Years	CK (U/L) 4–7 Years	CK (U/L) 7–13 Years	CK (U/L) 13–17 Years	25-OHD (ng/mL)
Male	<5	225–600	120–300	24–228	24–149	24–154	24–123	>30
Female	<5	225–600	120–300	24–228	24–149	24–247	24–270	>30

CRP—C-reactive protein; LDH—lactate dehydrogenase; 25-OHD—the major circulating form of vitamin D; CK—creatine kinase.

**Table 2:** Contingency tables and chi^2^ results.

	High CRP	Normal CRP	*p*	High LDH	Normal LDH	*p*	High CK	Normal CK	*p*
Respiratory symptoms	7	2	0.0399	6	2	<0.0001	2	5	0.7242
No respiratory symptoms	61	82		2	34		6	21	

CRP—C-reactive protein; LDH—lactate dehydrogenase; CK—creatin kinase.

**Table 3:** Clinical signs, CRP and LDH values of patients used for the using Spearman’s rank correlation test.

No.	CRP (mg/L)	LDH (U/L)	25-OHD	Clinical Signs
1	1.02	313	39.01	-
2	84.39	400	9.51	-
3	0.60	397	31.33	Pneumonia
4	7.90	404	22.17	-
5	25.29	354	25.64	Pneumonia
6	7.38	147	23.23	Pneumonia
7	18.91	224	31.33	-
8	16.38	273	18.13	-
9	7.18	265	34.78	-
10	30.39	265	24.55	-
11	0.60	162	62.75	-
12	9.84	237	26.11	-
13	10.56	287	29.63	-
14	24.20	250	27.95	Acute laryngitis
15	65.38	264	12.00	-
16	22.02	311	21.75	-
17	25.29	404	19.37	Pneumonia
18	22.60	183	30.94	Pneumonia
19	4.59	213	54.64	-
20	4.23	280	46.21	-
21	43.65	341	18.89	-
22	84.32	367	7.12	Acute laryngitis
23	2.69	304	35.65	-
24	76.31	257	17.07	-
25	24.33	260	29.29	-
26	1.49	228	46.51	Pneumonia
27	1.02	313	39.01	-

CRP—C-reactive protein; LDH—lactate dehydrogenase; 25-OHD—the major circulating form of vitamin D.

**Table 4:** The results of Spearman’s rank correlation test.

Spearman’s Rank Correlation Coefficient	Clinical Signs	CRP	LDH	25-OHD
Clinical signs	1	0.3958 (*p* = 0.0401)	0.5521 (*p* = 0.0028)	−0.3854 (*p* = 0.0471)
CRP	0.3958 (*p* = 0.0401)	1	0.2024 (*p* = 0.3114)	−0.8409 (*p* = <0.0001)
LDH	0.5521 (*p* = 0.0028)	0.2024 (*p* = 0.3114)	1	−0.3823 (*p* = 0.4904)
25-OHD	−0.3854 (*p* = 0.0471)	−0.8409 (*p* = <0.0001)	−0.3823 (*p* = 0.4904)	1

CRP—C-reactive protein; LDH—lactate dehydrogenase; 25-OHD—the major circulating form of vitamin D.

## Data Availability

Data available upon request.
